# Increased Sparsity of Hippocampal CA1 Neuronal Ensembles in a Mouse Model of Down Syndrome Assayed by Arc Expression

**DOI:** 10.3389/fncir.2017.00006

**Published:** 2017-02-03

**Authors:** Constance L. Smith-Hicks, Peiling Cai, Alena V. Savonenko, Roger H. Reeves, Paul F. Worley

**Affiliations:** ^1^Solomon H. Snyder Department of Neuroscience, Johns Hopkins University School of MedicineBaltimore, MD, USA; ^2^Department of Neurology, Johns Hopkins University School of MedicineBaltimore, MD, USA; ^3^The State Key Laboratory of Biotherapy, West-China Hospital, Sichuan UniversityChengdu, China; ^4^Department of Pathology, Johns Hopkins University School of MedicineBaltimore, MD, USA; ^5^Department of Physiology and Institute of Genetic Medicine, Johns Hopkins University, School of MedicineBaltimore, MD, USA

**Keywords:** Arc/Arg3.1, activity regulated cytoskeletal protein, neural-networks, catFISH, learning and memory

## Abstract

Down syndrome (DS) is the leading chromosomal cause of intellectual disability, yet the neural substrates of learning and memory deficits remain poorly understood. Here, we interrogate neural networks linked to learning and memory in a well-characterized model of DS, the Ts65Dn mouse. We report that Ts65Dn mice exhibit exploratory behavior that is not different from littermate wild-type (WT) controls yet behavioral activation of Arc mRNA transcription in pyramidal neurons of the CA1 region of the hippocampus is altered in Ts65Dn mice. In WT mice, a 5 min period of exploration of a novel environment resulted in Arc mRNA transcription in 39% of CA1 neurons. By contrast, the same period of exploration resulted in only ~20% of CA1 neurons transcribing Arc mRNA in Ts65Dn mice indicating increased sparsity of the behaviorally induced ensemble. Like WT mice the CA1 pyramidal neurons of Ts65Dn mice reactivated Arc transcription during a second exposure to the same environment 20 min after the first experience, but the size of the reactivated ensemble was only ~60% of that in WT mice. After repeated daily exposures there was a further decline in the size of the reactivated ensemble in Ts65Dn and a disruption of reactivation. Together these data demonstrate reduction in the size of the behaviorally induced network that expresses Arc in Ts65Dn mice and disruption of the long-term stability of the ensemble. We propose that these deficits in network formation and stability contribute to cognitive symptoms in DS.

## Introduction

Down syndrome (DS) results from the inheritance of three copies of chromosome 21 and is the leading chromosomal cause of intellectual disability. The Ts65Dn mouse model of DS is triploid for approximately half of the mouse orthologs of human chromosome 21 genes and recapitulates many aspects of the human condition including cognitive deficits and reduction of hippocampus dependent learning (Escorihuela et al., 1995; Reeves et al., 1995; Faizi et al., 2011). Electrophysiological evaluation of Ts65Dn hippocampal function reveals a reduction in synaptically evoked NMDA receptor responses at the Schaffer collateral synapse onto CA1 pyramidal neurons (Das et al., 2013), as well as reduced long-term potentiation (LTP) and increased NMDA-receptor long-term depression (LTD) of the Schaffer-CA1 synapse (Siarey et al., 1997, 1999; Kleschevnikov et al., 2004). The CA3 auto-associative network has been implicated in memory storage and retrieval (Bennett et al., 1994) and is altered in Ts65Dn mice (Hanson et al., 2007). In addition, hippocampal circuits in Ts65Dn mice exhibit an imbalance in excitation and inhibition with a shift towards greater inhibition, reviewed in Smith-Hicks (2013). The impact of these cellular and molecular changes on behaviorally induced neuronal networks is unknown.

Cellular immediate early genes (IEGs) are transcriptionally induced by patterned synaptic activity (Worley et al., 1993). The IEG *Arc* is induced by NMDA-dependent synaptic activity (Link et al., 1995; Lyford et al., 1995) and functions at excitatory synapses where it plays important roles in synaptic plasticity and memory consolidation (Shepherd et al., 2006; Wang et al., 2006; Park et al., 2008; Cao et al., 2015). *De novo* Arc mRNA can be detected at individual alleles in the nucleus of CA1 hippocampal neurons within minutes of initiating exploration of a new environment (Guzowski et al., 1999; Pevzner et al., 2012). Termination of Arc transcription is also rapid and associated with behavior such that within 20 min of return to the home cage, Arc mRNA is absent in the nucleus and is present in the cytoplasm and perinuclear region. A second behavioral experience can reinitiate *de novo* Arc transcription, and by combining two 5 min behavioral experiences in the same animal separated by 20 min in home cage it is possible to identify individual neurons that express Arc as a consequence of the first experience (Arc in cytoplasm/perinuclear) or the second experience (Arc in nucleus). The behavioral dependence and percentage of responding neurons support the notion that Arc mRNA is expressed in hippocampal place cells (Guzowski et al., 2004, 2006; Witharana et al., 2016). Precedent from *in vivo* recordings of hippocampal place cells predicts that Arc mRNA will be present in the cytoplasm and nucleus of the same neurons if both experiences are in the same environment; and this has been confirmed in several behavioral paradigms (Guzowski et al., 1999, 2005, 2006; Burke et al., 2005; Chawla et al., 2005; Miyashita et al., 2009; Kubik et al., 2012; Pevzner et al., 2012). This approach, termed catFISH (cellular compartment analysis of temporal activity by Fluorescence *in situ* Hybridization), can be used to assess the identity, reliability and number of neurons that are activated during a behavioral experience.

Here, we monitor Arc mRNA transcription to assess hippocampal pyramidal cell properties in the Ts65Dn mouse model of trisomy 21. Hippocampal pyramidal neuron numbers in Ts65Dn mice are not different than wild-type (WT; Insausti et al., 1998; Lorenzi and Reeves, 2006) so measures of percentages of neurons can be directly compared. We report that Arc transcription is induced in a smaller percentage of CA1 pyramidal neurons, indicating greater sparsity, in Ts65Dn mice than WT controls. Despite the greater sparsity, neurons that expressed Arc in the first experience were reactivated to express Arc mRNA upon re-entry into the same environment after 20 min. However, this pattern of reactivation was disrupted when Ts65Dn mice had several preceding daily exposures to the same environment. These observations reveal changes in the recruitment of neurons and stabilization of behaviorally linked networks as a contributing factor for memory deficits in the mouse model of trisomy 21.

## Materials and Methods

### Animals

Founder B6EiC3H-a/A-Ts65Dn (Ts65Dn) mice were obtained from the Jackson Laboratory and maintained as an advanced intercross on a (C57BL/6J × aC3H/HeJ)*F*_n_ background. Male mice were used in these experiments and WT littermates served as controls. Mice homozygous for a Pde6b mutation causing blindness were excluded from analysis. This study was carried out in accordance with the recommendations of Johns Hopkins Institutional Animal Care and Use Committee and they approved all animal care and experimental procedures.

### Behavior

A total of 33 2–4 month old male mice were single housed and handled daily for 5 days to familiarize them to the examiner and handling. Environment “A” was a 40 cm sq box with novel objects at fixed positions on the floor of the box and along one wall of the box. Behavioral phenotyping was performed using Any Maze v5.14 (Stoelting Co.) on eight male mice, four from each genotype.

### Fluorescence-*In Situ* Hybridization

Mice were sacrificed following 30 s exposure to 85% isoflurane by decapitation. *in situ* hybridization was performed as previously described (Guzowski et al., 1999). Briefly, brains were rapidly removed and quick-frozen in a beaker of isopentane equilibrated in dry ice/ethanol slurry and stored at −80°C until further processing. A commercial transcription kit (MaxiScript; Ambion, Austin, Texas) and premixed RNA labeling nucleotide mix containing fluorescein-labeled UTP (Roche Molecular Biochemicals) was used to generate the cRNA riboprobe. The riboprobes were purified on G-50 spin columns (Pharmacia) and the yield and integrity was confirmed by gel electrophoresis. The plasmid used to generate the *Arc* antisense and sense riboprobes contained a previously described, nearly full-length cDNA (~3 kbp) of the *Arc* transcript (Lyford et al., 1995). Coronal brain sections (20 μm) containing the dorsal hippocampus were prepared using a cryostat and arranged on slides (Superfrost Plus, VWR) so that all experimental groups were represented on single slides. Slide mounted brain sections were air dried and stored frozen at −20°C until use when they were fixed in 4% buffered paraformaldehyde, treated with 0.5% acetic anhydride/1.5% triethanolamine, and equilibrated in 2× SSC. Slides were then incubated in 1× prehybridization buffer (Sigma) for 30 min at room temperature. Arc/Arg3.1 riboprobe labeled with Fluorescein-UTP (100 ng) was diluted to 100 μl in a commercial hybridization buffer (Amersham), heat denatured, chilled on ice, and then added to each slide. Hybridization was carried out at 56°C for 16 h. Slides were then washed to a final stringency of 0.5 × SSC at 56°C. Endogenous peroxidase activity was quenched with 2% H_2_0_2_ in PBS, slides were incubated with the horseradish peroxidase (HRP)-antibody conjugate (Roche Molecular Biochemicals) 2 h at room temperature and then washed three times in Tris-buffered saline (with 0.05% Tween-20). The peroxidase conjugate was detected using FITC-TSA fluorescence system (PerkinElmer Life Sciences) and counterstained with DAPI. Slides were cover-slipped with antifade media (Vectashield; Vector Labs, Burlington, CA, USA) and sealed.

### Imaging, Cell Counting and Analysis

Imaging, cell counting and analysis were performed according to the protocol summarized in the technique article (Guzowski and Worley, 2001) and briefly outlined here. Stained slides were analyzed using a Zeiss LSM 510 confocal microscope. PMT assignments, pinhole sizes and contrast values were kept constant across all confocal sessions. The regions of interest were *z* sectioned in 1.0-micron optical sections. Each cell was characterized through several serial sections, and only cells containing whole nuclei were included in the analysis. An experimenter blinded to the behavioral conditions and genotype performed all analyses. The DAPI stain revealed nuclei of two distinct morphologies. The majority of cells in the CA1 layer had large diffusely stained nuclei that were presumed to be neurons. Only these cells were included in the analysis. The remainder of the cells had much smaller nuclei that were stained strongly with DAPI and were thought to be glial. Arc was previously shown to be induced by behavior or seizure in the hippocampus, primary somatosensory cortex and dorsal striatum of rats and co-localized with neuronal (NeuN-positive) and not with glial (GFAP-positive) cells (Vazdarjanova et al., 2006). In addition, Arc was found exclusively in non-GABAergic α-CaMKII-positive hippocampal and neocortical neurons of rats that had explored a novel environment (Vazdarjanova et al., 2006). The assignment of nucleus vs. cytoplasm/perinuclear signal was made by first selecting the pyramidal cell layer by visualizing only the color channel containing the DAPI information. Using the “Lasso” tool the pyramidal cell nuclei were encircled. The DAPI channel was then turned off and the fluorescein channel turned on. The “intranuclear-foci positive” designation was given to circles (nuclei) that contained one or two intense areas of fluorescein label, and the designation “cytoplasmic positive” was given to circles (nuclei) that contained perinuclear fluorescein labeling (Guzowski et al., 1999; Guzowski and Worley, 2001). The assignment of cells as negative or positive for Arc was made only after viewing multiple optical sections that contained each individual cell; this method improved accurate assignment and reduced counting errors.

Similarity score is a measure used to combine the cell staining data from four groups corresponding to the number of neurons that are Arc negative (α), Arc in nucleus only (β), Arc in cytoplasm/perinuclear area only (γ), and Arc in both nucleus and cytoplasm/perinuclear (δ), into a single value that can be used to compare cell activity across different animals (Vazdarjanova and Guzowski, 2004). The similarity score with a value of 1 represents a single neuronal population that was reliably reactivated, while a value of 0 indicates that two statistically independent populations were activated during consecutive exploratory experiences. The similarity score is derived from the following calculations:

1. Percentage of neurons activated by exploration #1, E1 = (γ + δ)/(α + β + γ + δ).
2. Percentage of neurons activated by exploration #2, E2 = (β + δ)/(α + β + γ + δ).
3. The probability that the neurons activated by both experiences are independent is p(E1E2)= E1 × E2.
4. The probability that there is some overlap in the populations activated by both experiences: pO(E1E2) = δ − (E1E2). This is a measure of deviation from the independence hypothesis.
5. Least behavior = the smaller of the populations activated by exploration 1 or exploration 2.
6. Similarity score = pO(E1E2)/(least behavior−p(E1E2). This normalizes the pO(E1E2) fraction to a perfect A/A condition. Where a perfect A/A is 1, and a perfect A/B is 0.

### Statistics

Analyses of cellular expression of Arc were performed with GraphPad Prism 6.0. Data are represented as mean ± SEM. Two-way analysis of variance (ANOVA) followed by *post hoc* analysis using Tukey’s, Sidak multiple comparison tests were performed as indicated. The analyses of behavioral data were performed using Statistica 13.0 (Dell Inc., Tulsa, OK, USA). The data was presented as mean ± SEM. A two-way mixed design ANOVA followed by Fisher’s Least Significant Difference (LSD) *post hoc* analysis was performed when indicated. The null hypotheses were rejected at a 0.05 level of significance.

## Results

### Wild-Type and Ts65Dn Mice Exhibit Similar Exploratory Behavior in a Novel Environment

**Figure 1:**
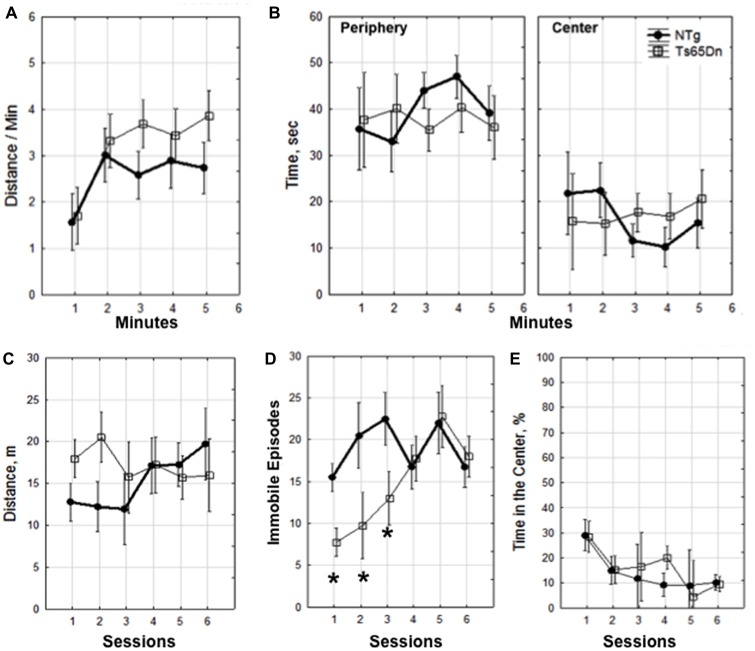
**Dynamics of exploration.** Within session 1 **(A,B)**. **(A)** Distance traveled per minute and **(B)** time in seconds spent in the periphery and center. Dynamics of exploration across sessions 1–6 **(C–E)**. **(C)** Distance traveled in meters, **(D)** episodes of immobility, *p < 0.05. **(E)** percentage of time spent in the center of the environment. The data is presented as mean ± standard error of the mean (SEM). Four mice of each genotype were used for the behavioral analysis.

We compared the exploratory behavior of WT and Ts65Dn male mice. Mice explored a novel environment once a day for four consecutive days and on day 5 they explored the environment two times separated by 20 min. Using a two-way mixed design ANOVA we show that there was no significant difference between the Ts65Dn and WT mice in the distance traveled per minute (*p* = 0.6), time spent exploring the periphery or center of the box in the first session (*p* = 0.9; Figures 1A,B). In addition, there were no genotype-related differences in the distance traveled across sessions (*p* = 0.17; Figure 1C). Noteworthy, mice of both genotypes did not decrease exploratory activity within or between sessions (Figures 1A,C) indicating a lack of habituation and continuous motivation to explore in this enriched environment. We also analyzed episodes of immobility across sessions using a two-way mixed design ANOVA and showed an interaction between genotype and session (*F*_(5,30)_ = 4.85, *p* < 0.003). LSD *post hoc* analysis revealed that Ts65Dn mice had fewer episodes of immobility on days 1–3, *p* < 0.05 (Figure 1D). This difference indicates that during the first 3 sessions Ts65Dn mice were making less short stops while ambulating. Fewer number of stops during these sessions was consistent with slightly longer distances covered during exploration (Figure 1C) however this tendency did not reach significance. There was also no difference in indexes of anxiety (the percentage of time spent in the center of the environment) when compared across sessions (*p* = 0.4; Figure 1E).

### Fewer Ts65Dn CA1 Hippocampal Neurons Induce Arc Transcription during Exploratory Behavior in a Novel Environment when Compared to Wild-Type Mice

**Figure 2:**
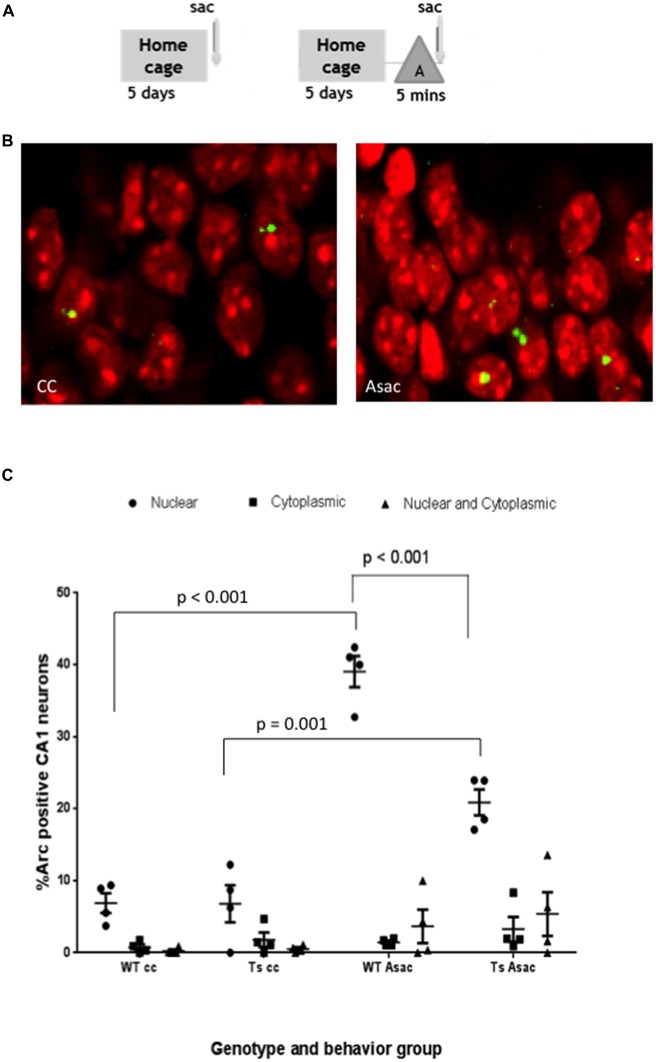
**Pyramidal neurons in the CA1 hippocampal region of Ts65Dn mice express Arc mRNA in response to exploration of a novel environment but the percent of neurons expressing Arc is reduced compared to wild-type (WT) CA1. (A)** Schematic of behavioral paradigm. Mice were taken from their home cage and immediately sacrificed (cage control, cc), or allowed to explore a novel enriched environment “A” for 5 min and then immediately sacrificed (Asac). **(B)** Representative confocal images from each behavioral condition showing transcription foci of Arc mRNA (yellow) within the nucleus (red). **(C)** Scatter plot showing the mean percent ± SEM of Arc mRNA positive neurons in area CA1 of WT and Ts65Dn (Ts) mice. The percent of neurons with intranuclear foci of Arc mRNA is greater in the Asac group compared to cc for both WT and Ts65Dn mice (WT *p* < 0.001, Ts *p* = 0.001). The percentage of neurons with intranuclear foci of Arc mRNA in the Ts65Dn group is reduced compared to WT mice, *p* < 0.001. Four mice per genotype per group were used. Neurons counted per group were WT cc – 900, Ts cc – 340, WT Asac – 710, Ts Asac – 767.

Ts65Dn (Ts) mice and age-matched WT controls were allowed to explore a novel environment “A” for 5 min and then immediately sacrificed (Asac). Behavioral controls (caged control; cc) from each group were sacrificed immediately upon removal from the home cage (Figure 2A). To assure that mice were motivated to explore their environment, mice were food restricted to achieve 80% of their initial body weight and allowed to forage for randomly dispersed pellets during each exploratory epoch in a spatially enriched environment (Kentros et al., 2004). Time course studies in rats indicate that 30 s of exploration is sufficient to activate Arc transcription in an ensemble of CA1 neurons that matches the predicted sparsity from electrophysiological recordings (Pevzner et al., 2012). We observed Arc RNA in the nucleus, shown as yellow puncta in representative confocal images (Figure 2B) in 39.05 + 2.16% of pyramidal neurons from the WT Asac CA1 as compared to 6.89 + 1.35% in the WT cc group (*p* < 0.01, mean 32.16, 95% CI 38.68–25.63) (Figure 2C). When the same experiment was performed using Ts65Dn mice, 20.86 + 1.79% of CA1 Asac neurons expressed Arc mRNA in the nucleus compared to 6.79 + 2.57% in the Ts cc group (*p* < 0.01, mean difference 14.06 and 95% CI 20.59–7.53). Comparison of responses revealed that a reduced percentage of neurons transcribed Arc in Ts65Dn compared to wild type mice. A two-way ANOVA found a main effect of genotype, *F*_(3,36)_ = 34.47, *p* < 0.001; a main effect of cellular localization, *F*_(2,36)_ = 120.3, *p* < 0.001; and an interaction between genotype and cellular localization *F*_(6,36)_ = 23.72, *p* < 0.001. Tukey’s *post hoc* comparison between WT and Ts Asac groups revealed a significant decrease in the percentage of Ts65Dn neurons expressing Arc mRNA in the nucleus (*p* value < 0.001; mean difference 18.20; 95% CI = 11.67–24.72).

### Reactivation of Arc in CA1 Pyramidal Neurons Is Preserved in Ts65Dn Mice during a Second Exploration of the Same Environment within 20 min

**Figure 3:**
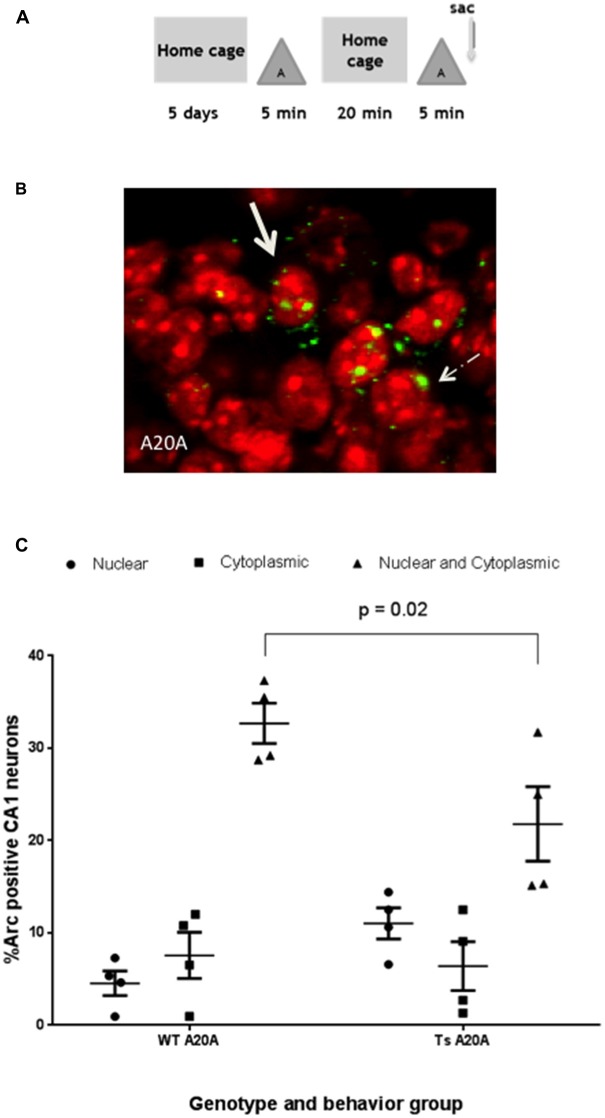
**WT and Ts65Dn mice reactivate Arc transcription in individual CA1 neurons in response to sequential 5 min epochs in the same environment separated by 20 min. (A)** Schematic of the behavioral paradigm. Mice were taken from their home cage and allowed to explore a novel environment “A” for 5 min then returned to their home cage for 20 min. They were then allowed to re-explore environment “A” for 5 min and were then immediately sacrificed (A20A). **(B)** A neuron containing a single focus of Arc mRNA in the nucleus is indicated by the arrow with dashed line. A solid arrow identifies a neuron with Arc mRNA in the nucleus (red) and cytoplasmic (perinuclear). **(C)** The percentage of Arc positive neurons for each staining profile: nucleus only, cytoplasmic only or both nuclear and cytoplasmic region is represented in scatter plots showing mean percent ± SEM. In Ts65Dn fewer neurons are double positive for Arc mRNA in both nucleus and cytoplasm as compared to WT CA1; *p* = 0.02. *N* = 4 mice per genotype; 583 WT neurons and 423 Ts65Dn neurons were counted.

As a further test of hippocampal functionality, Ts65Dn and WT mice were tested for Arc induction in CA1 neurons in response to two sequential experiences in the same environment separated by 20 min (Figure 3A). Arc mRNA in the cytoplasm identified neurons that induced Arc transcription in the first experience, while Arc mRNA in the nucleus identified neurons that induced Arc in the second experience. Arc mRNA in both nucleus and cytoplasm identified neurons that were reactivated and induced Arc transcription during both experiences (Figure 3B). In WT CA1 neurons, 32.7 + 2.18% were reactivated and expressed Arc mRNA in the cytoplasm and nucleus, while 4.54 + 1.32% expressed Arc in the nucleus only and 7.53 + 2.5% in the cytoplasm only (Figure 3C). We used these numbers to calculate a “similarity score” (Vazdarjanova and Guzowski, 2004), which provides an index of the fidelity of reactivation (see “Materials and Methods” Section). The similarity score of 0.88 is consistent with a high degree of re-activation in this experimental paradigm.

In Ts65Dn CA1 neurons, 21.8 + 4.03% of neurons are reactivated and express Arc mRNA in the cytoplasm and nucleus, while 11.03 + 1.67% expressed Arc in the nucleus and 6.40 + 2.65% in the cytoplasm only. The percentage of neurons that are reactivated is similar to the percentage of neurons that induce Arc transcription during a single A experience (20.86 + 1.79%), however the reactivated neurons in Ts65Dn mice is 60% of the size of the reactivated network in WT mice. Comparison of Ts65Dn and WT in a two-way ANOVA revealed significant interaction between genotype and cellular localization, *F*_(2,18)_ = 5.86, *P* = 0.01; a main effect of cellular localization, *F*_(2,18)_ = 40.73, *P* < 0.001 and no effect of genotype, *F*_(1,18)_ = 0.79, *P* = 0.38. Sidak’s *post hoc* analysis reveals that the percent of neurons that are reactivated and express Arc mRNA in both nucleus and cytoplasm of Ts65Dn mice is less than WT (*p* = 0.02, mean difference 10.90, 95% CI 1.438–20.37). Nevertheless, the calculated similarity score of 0.78 in Ts65Dn mice is not significantly different than WT (0.88) indicating that CA1 neurons activated in the first experience are effectively reactivated in the second experience in the same enriched environment. Thus, despite increased sparsity findings suggest that plasticity mechanisms required to maintain the CA1 pyramidal cell ensemble for 20 min are preserved in Ts65Dn mice.

### Daily Repetition of Behavioral Experience Disrupts Subsequent Arc Reactivation in CA1 Pyramidal Neurons of Ts65Dn Mice

**Figure 4:**
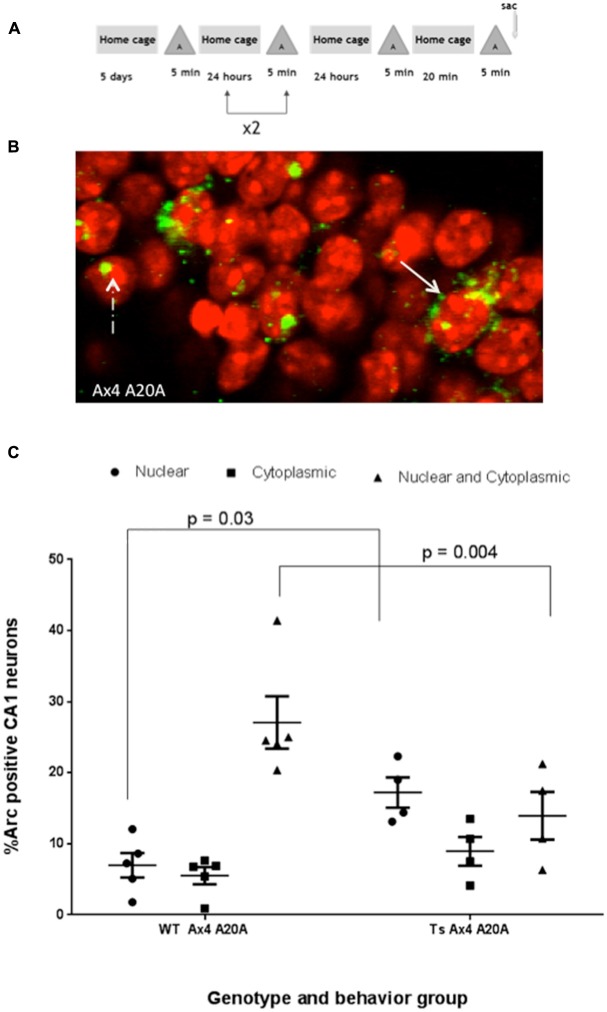
**Repeated daily exposure to enriched environment “A” disrupts fidelity of reactivation in CA1 of Ts65Dn mice. (A)** Behavioral timeline. **(B)** Solid arrow indicates double positive neurons expressing Arc transcript in nucleus and cytoplasm, while dashed arrow indicates neuron with nuclear only Arc transcript. **(C)** Scatter plot showing the mean percent ± SEM of Arc positive neurons in area CA1 of WT and Ts65Dn (Ts) mice. The percent of neurons with intranuclear Arc foci is significantly greater in Ts65Dn CA1, *p* = 0.03, while fewer Ts65Dn neurons are double positive for Arc, *p* = 0.004. Five WT mice with a total of 656 neurons were counted and 4 Ts65Dn mice with a total of 495 neurons were counted.

The percentage of CA1 neurons that express Arc mRNA or that reactivate Arc transcription in a second experience remains stable in rats despite repeated daily exposure to the same environment indicating that Arc transcription is not dependent on novelty, and is similar in this regard to place cells (Guzowski et al., 2006). To assess how repeated experience might influence CA1 neurons activated during exploration, Ts65Dn and WT mice were allowed to explore the same enriched environment once per day for four consecutive days. On the fifth day mice were allowed to explore the same environment twice for 5 min separated by 20 min (Ax4 A20A Figure 4A). Arc transcription occurs despite repeated daily exploration. Arc in the nucleus represents neurons that were active in the second exploration only and Arc in both nucleus and cytoplasm represents neurons that were reactivated and expressed Arc during both explorations on day 5 (Figure 4B). In WT mice this daily repetition did not alter the total percentage of Arc-positive CA1 pyramidal neurons (nucleus only + cytoplasm only + both nucleus and cytoplasm) that expressed Arc in each of the three paradigms (Asac vs. A20A vs. Ax4 A20A (44.1% vs. 44.7% vs. 39.6%)). The percentage of neurons showing reactivation of Arc mRNA transcripts in the Ax4 A20A group was not different from that of the A20A paradigm (27.09 + 3.68% vs. 32.7 + 2.18%). Moreover, the similarity score of 0.86 for the Ax4 A20A group was not different from the A20A (0.88).

**Figure 5:**
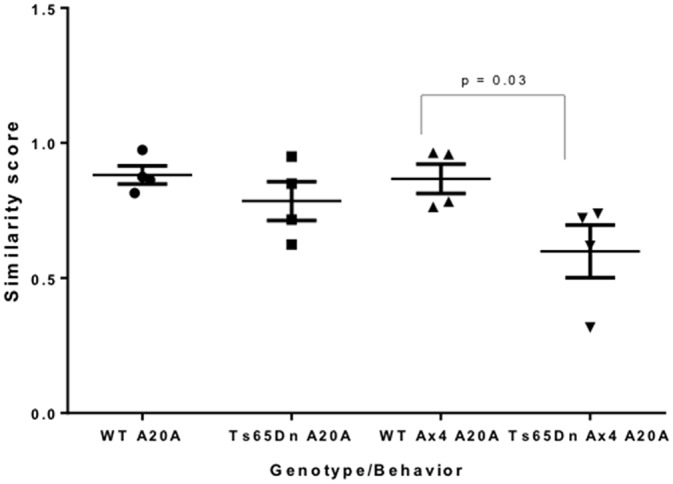
**Similarity scores of CA1 neurons in A20A and Ax4 A20A behavioral groups in Ts65Dn and WT mice.** Data is represented by scatter plots of the mean ± SEM. A similarity score of 1 indicates complete overlap of neurons expressing Arc mRNA following the first and second exploration, while a similarity score of 0 indicates no overlap. The similarity score of 0.59 in CA1 of Ts65Dn Ax4 A20A mice is significantly smaller compared to WT Ax4 A20A mice (*p* = 0.03).

In Ts65Dn mice, daily repetition did not alter the total percentage of Arc-positive CA1 pyramidal neurons (nucleus only + cytoplasm only + both nucleus and cytoplasm) compared to Ts65Dn A20A (A20A 39.2% vs. Ax4 A20A 40.12%) and this was not significantly different than WT mice (A20A 44.70% vs. Ax4 A20A 39.60%). However, the percentage of CA1 neurons that were reactivate in Ts65Dn mice during exploration in the Ax4 A20A paradigm was 13.95 + 3.35% compared to 27.09% + 3.68% in WT. Two-way comparison of Arc response between WT and Ts65Dn in the Ax4 A20A group found an interaction between genotype and cellular localization *F*_(2,21)_ = 11.31, *P* < 0.001, a main effect of cellular localization *F*_(2,21)_ = 14.11, *P* < 0.001, and no effect of genotype *F*_(1,21)_ = 0.007, *P* = 0.93. *Post hoc* analysis revealed a significantly smaller population of reactivated neurons in Ts65Dn compared to WT (mean difference 13.14, 95% CI 3.87–22.42, *p* = 0.004; Figure 4C). Of note, the population of neurons that expressed Arc mRNA only during the second experience was significantly increased in the Ts65Dn CA1 vs. WT (Ts65Dn 17.22 + 2.12%; WT 6.98 + 1.72%; *p* = 0.03; Figure 4C). These differences are captured in the similarity score 0.59 in Ts65Dn mice for the Ax4 A20A behavioral group. Two-way ANOVA was performed comparing similarity scores for the WT and Ts65Dn, A20A and Ax4 A20A groups and showed a main effect of genotype *F*_(1,12)_ = 7.086, *p* = 0.02; no effect of behavior *F*_(1,12)_ = 2.138, *p* = 0.16; and no interaction between genotype and behavior *F*_(1,12)_ = 1.564, *p* = 0.2. Sidak’s *post hoc* analysis performed between the WT and Ts65Dn A20A groups; and the WT and Ts65Dn Ax4 A20A groups revealed no difference for the A20A group but a mean difference of 0.27, 95% CI 0.02–0.52, *p* = 0.03 for the Ax4 A20A paradigm (Figure 5). Accordingly, repeated daily experience results in reduced fidelity of reactivation of CA1 neurons in Ts65Dn Ax4 A20A. This contrasts with preserved fidelity of reactivation in Ts65Dn over 20 min and suggests failure of mechanisms that mediate long-term training dependent plasticity.

## Discussion

The present study identifies alterations of *in vivo* hippocampal function that can be linked to hippocampal learning deficits in the Ts65Dn model of DS. A primary finding is that fewer CA1 pyramidal cells express Arc mRNA after the first experience exploring an enriched novel environment. One can hypothesize that this observation could be the result of different exposure of mutant and WT mice to the environment. To test for this possibility we performed detailed behavioral analyses during the first and repeated exposures to the enriched environment. Exploratory behaviors were similar between genotypes with the Ts65Dn mice showing a tendency for longer distances traveled while exploring the environment and making fewer short stops than WT controls. Measures of thigmotaxis revealed no genotype-related differences in levels of anxiety. The results of behavioral analyses are consistent with published data reporting that the Ts65Dn mice show normal or hyperactive exploration in the open filed (Coussons-Read and Crnic, 1996; Martínez-Cué et al., 2002; Faizi et al., 2011; Incerti et al., 2011). In our enriched environment the Ts65Dn mice explored as actively as control mice with some measures indicating a slight hyperactivity. These behavioral results make it highly unlikely that the reduced exploration of environment is the cause of deficient engagement of Arc-dependent plasticity in CA1 pyramidal cells in the Ts65Dn mice. Although we did not directly measure neuronal activity the present observations demonstrate that the percent of neurons that are capable of engaging Arc-dependent plasticity mechanisms is reduced in the Ts65Dn model of trisomy 21. This is an especially meaningful indication of altered plasticity since Arc protein expression is required for the formation of stable neuronal ensembles during learning (Cao et al., 2015).

To our knowledge, increased sparsity has not been previously reported in a model of cognitive dysfunction. Determinants of sparsity remain a focus of investigation (Witharana et al., 2016). Sparsity is highly consistent between different animals of the same genotype and closely similar in CA1 hippocampal neurons between mouse and rat. The observation that sparsity can be markedly different in different neural structures, for example granule cell of the dentate gyrus vs. CA neurons of the hippocampus, suggests that sparsity is defined, in part, by the intrinsic architecture of synaptic connections. Although the number of CA1 neurons in Ts65Dn mice between the ages of 2–6 months old is not significantly different from wild type (Insausti et al., 1998; Lorenzi and Reeves, 2006) the fine structure of synaptic connectivity in Ts65Dn mice is not known. Ts65Dn mice exhibit an imbalance between excitation and inhibition that is shifted towards heightened inhibition (Best et al., 2012; Kleschevnikov et al., 2012). This may limit the ability of pyramidal neurons to respond to patterned synaptic activity and so contribute to increased sparsity. Alternative mechanisms may include differences in synaptic plasticity mechanisms or epigenetic silencing of Arc. Hypermethylation of Arc is associated with schizophrenia (Chuang et al., 2016) and is reported to occur 24 h after electroconvulsive shock (Dyrvig et al., 2012). Although the methylation status of Arc in DS is not known, DNA methyltransferase3L is triplicated on chromosome 21 and global hypermethylation is reported in the DS fetal cortex (Lu et al., 2016).

A second notable finding in Ts65Dn mice is deterioration of the ability to reactivate a CA1 ensemble during a second experience in the same enriched environment. This phenotype emerges in Ts65Dn mice upon daily repeated exposure to the enriched environment, and contrasts with the preserved ability of Ts65Dn to reactivate Arc mRNA expression when there is one prior exposure to the environment. The disruptive effect of prior exposure is unexpected since daily repetition typically stabilizes CA1 pyramidal cell representations in WT animals (Eichenbaum et al., 1999; Guzowski et al., 2004). Long-term but not short term stability of place cell networks is disrupted by deletion of the transcription factor zif268/erg1 (Renaudineau et al., 2009) which contributes to Arc transcriptional regulation (Penke et al., 2011). These combined observations suggest that Ts65Dn exhibit a relative preservation of short-term plasticity mechanisms that acutely stabilize ensembles, but exhibit disruption of long-term plasticity mechanisms that establish stable pyramidal cell representations. Our findings focus attention on determinates of sparsity and learning-dependent ensemble stabilization as a basis for cognitive deficits in DS.

## Author Contributions

CLS-H, PFW and RHR conceived the experiments. CLS-H designed the experiments, performed a portion of the study and supervised the project. PC performed the analysis of the data. AVS. performed and analyzed the behavioral data. All authors contributed to the interpretation and writing of the manuscript. PFW provided overall supervision.

## Conflict of Interest Statement

The authors declare that the research was conducted in the absence of any commercial or financial relationships that could be construed as a potential conflict of interest.
